# 

**Published:** 1871-03

**Authors:** 


### THE GEORGIA MEDICAL ASSOCIATION.

The annual meeting of the Georgia Medical Association will be held on the 12th of April next, at Americus, Ga. Our friends of Americus assure us the profession will be welcomed to their city with open arms, which no one will doubt, when it is remembered that it is the home of Drs. Cooper, Greene, Hinkle, Hawkins, Hudson and others—well known for their talents and Christian characters.

It would seem a useless waste of time to urge upon the profession the great importance of attending the annual sessions of its State Association. There never has been a time when the profession could safely neglect the assembling of its members to consult upon and adopt measures for the advancement of science, and for elevating the standard of professional morality. Now, more than ever, under the demoralization of the times—when avarice pervades all ranks of society—the union of good men, true to principle, and jealous of the old "landmarks," should strive to rescue the profession from pollution and from its fearful sweep to chaos and ruin. We therefore entreat—we implore—the members of the Association and all others who may desire to become such, and are eligible, to think of these things—to weigh the value of the vast interests at stake, and to attend the next meeting of the Association.

In this connection we beg leave to return our thanks to the corresponding Secretary of the Association for his kindness in forwarding us the transactions of the last two annual meetings of that body.

### AMERICAN MEDICAL ASSOCIATION.

The twenty-second annual session of the Association will be held in San Francisco, California, May 20, 1871, at 11 A. M. Some of the distinguished men of the profession, of the different States, have been appointed on various Committees, and are expected to report. Among this number, we notice the names of Dr. Juriah Harriss, of Savannah, Ga., and Dr. R. F. Michel, of Montgomery, Ala. We trust that every State will be largely represented in this Congress—the great central brotherhood—of the profession.

We have received from Dr. Wm. B. Atkinson, the permanent Secretary, a notice of this meeting, embracing a list of the names of the various committees. We tender him our thanks. Dr. Atkinson, as permanent Secretary, has shown an earnestness and zeal in his work, as creditable to him, as it has been beneficial to the profession. Long may he live to occupy the position.

### OUR EXCHANGES.

*The American Practitioner :* A Monthly Journal of Medicine and Surgery: Edited by David W. Yandell, M. D., Professor of Clinical Surgery, in the University of Louisville, and Theophilus Parvin, M. D., Professor of the Medical and Surgical Diseases of Women, in the University of Louisville. This Journal is one of the best published. Its columns are weighted with valuable medical articles. To say that it is ably edited, it is only necessary to refer to the distinguished names of Yandell and Parvin. The typographical execution of the Practitioner is par excellence —a model of beauty and neatness. Published by John P. Morton & Co., Louisville, Ky. Terms, $3.00 per annum.

We have received many journals, books and pamphlets, which, for want of space, we have been forced, reluctantly, to omit to notice in this number. We beg to state that we shall not fail to notice, at as early a date as possible, the various journals, books, &c., sent us. We are under lasting obligations to our friends for the kindness shown us, and shall pay the debt, with interest, we hope, very soon. Whenever we fail in those courtesies due our professional brethren, they may know that want of space, and no lack of appreciation of their kindness, is the reason.

